# A rare case of cancer-to-cancer metastasis: breast cancer to renal cell cancer: Case report and review of literature

**DOI:** 10.1007/s10354-019-0694-y

**Published:** 2019-04-30

**Authors:** Ioana-Claudia Lakovschek, Edgar Petru, Marion J. Pollheimer, Manfred Ratschek, Herbert Augustin, Vesna Bjelic-Radisic

**Affiliations:** 1 Department of Obstetrics and Gynecology, Division of Gynecology, Medical University of Graz, Auenbruggerplatz 14, 8036 Graz, Austria; 2 Institute of Pathology, Medical University of Graz, Graz, Austria; 3 Department of Urology, Medical University of Graz, Graz, Austria

**Keywords:** Breast cancer, Tumor-to-tumor metastasis, Renal carcinoma, Synchronous cancer, Bilateral breast cancer

## Abstract

**Background:**

Cancer-to-cancer metastasis is very rare with less than 50 cases described in literature. This article reports a case of breast cancer with synchronous metastasis to clear cell renal cell cancer.

**Case description:**

A 79-year-old woman was diagnosed with a bilateral breast carcinoma. Sonographic staging investigation of the abdomen revealed a 6 cm wide expansion of the right kidney. Bilateral mastectomy and nephrectomy of the right kidney was performed. The histology revealed a clear cell renal cell carcinoma and in the center of the tumor a 0.5 cm metastasis of the breast cancer. The patient’s comorbidities and performance status precluded chemotherapy und she received palliative radiotherapy, targeted monoclonal antibody therapy and antihormonal treatment.

**Conclusions:**

Even if cancer-to-cancer metastasis is a very rare phenomenon, the simultaneous or consecutive finding of a renal tumor in women with breast cancer should be carefully evaluated.

## Introduction

In contrast to synchronous occurrence of cancer which is found in up to 8% of cases, cancer-to-cancer metastasis is very rare with less than 50 cases described in the literature [1]. This article reports a case of breast cancer with synchronous metastasis to a clear cell renal cell cancer (RCC). To the best of our knowledge, there were only six other cases of breast cancer metastasis to an RCC with a diagnosis in lifetime and only two of them with synchronous appearance. Since the RCC often occurs as a comalignancy and it is also a good recipient of metastases, the simultaneous finding of a renal tumor in women with breast cancer should be carefully evaluated [2, 3].

## Case description

**Fig. 1:**
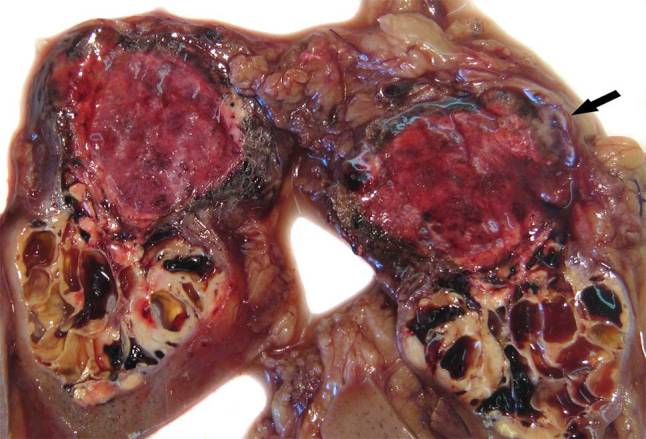
Primary renal cell cancer with synchronous metastasis from breast cancer (*arrow*)

A 79-year-old woman presented with palpable tumors in both breasts. Mammography strongly suggested bilateral multicentric breast cancer with a large lesion in the right breast of 4 cm and a 2.5 cm lesion in the left breast. Breast biopsies showed bilateral high grade (G2/3) invasive ductal breast cancer. Staging examination, chest X‑ray and bone scintigraphy showed no evidence of metastases but abdominal sonography revealed a 6 cm mass of the right kidney. Computed tomography suggested an RCC. Because of reduced performance status (multiple comorbidities) and the patient’s wish, chemotherapy was not reasonable. Bilateral mastectomy with bilateral sentinel node biopsy and dissection of the right axilla was performed. Final histology showed multicentric invasive ductal cancer of the right breast (T2N3M0, G3). Immunohistochemical staining revealed the following status of the tumor: estrogen receptors (ER) were 70% positive, progesterone receptors (PR) were 10% positive, the status of human epidermal growth factor receptor 2 (HER-2) was highly positive and the proliferation marker Ki67 also showed a high positivity of 80%. In the left breast an invasive ductal cancer was also diagnosed (T2N0M0, G2, ER 80%, PR 70%, HER-2 highly positive and Ki67 70%). Right nephrectomy was performed and histology showed a 6.5 cm RCC (T3aN0M0, G2). The exact examination of the tumor revealed in the center of the tumor a 0.5 cm metastasis from the invasive ductal breast cancer (Fig. 1).

**Fig. 2:**
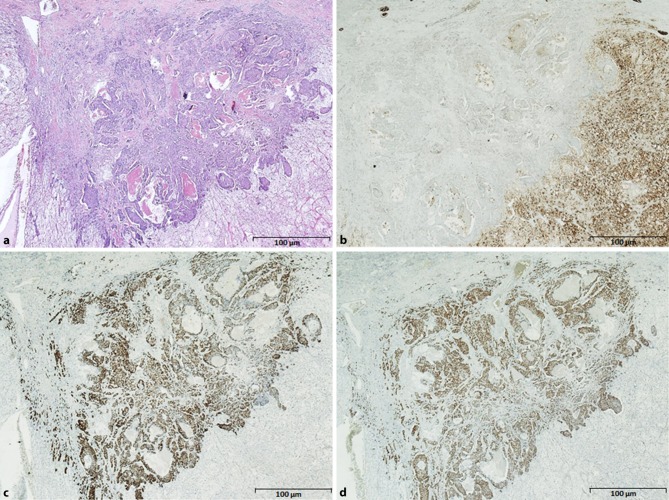
Histological section of the clear renal cell carcinoma with metastasis from breast cancer. **a** HE: hematoxilin eosin, **b** CD10: marker for renal cell carcinoma, **c** ER: estrogen receptor and **d** GATA-3: marker for breast cancer

Immunohistochemistry (GATA-3 and ER markers were positive) confirmed breast cancer metastatic to the RCC (Fig. 2a–d).

The diagnose of metastatic breast disease was established and the patient received trastuzumab, anastrozole and irradiation of the right chest wall as well as the supraclavicular region as palliative therapy. The patient reported pain in the cervical spine and right shoulder 8 months after diagnosis. Bone scintigraphy revealed a new metastasis of the right humerus. The patient subsequently received palliative radiotherapy of the humerus, fulvestrant and denosumab. Worsening of chronic heart failure and subsequent rapid deterioration of the patient’s general health status led to her death 3 years after diagnosis of breast cancer and in almost stable conditions of cancer disease.

## Conclusion

Synchronous multiple cancers are defined as a cancer diagnosed simultaneously with another cancer or within a time frame of 6 months. The presence of two synchronous cancers in one patient is not an uncommon scenario. The risk for a second synchronous cancer at diagnosis of breast cancer is approximately 2–3%. The most common synchronous and metasynchronous malignancies after breast cancer are malignancies of the thyroid gland and the female genital tract [4]. Synchronous cancers of the breast and the kidney occur occasionally. There are no common risk factors in carcinogenesis, except patient age. Increased detection of such synchronous and concomitant cancers may also be due to improved imaging, biochemical work-up and increased life expectancy. Sonography of the abdomen can yield incidental diagnoses of abdominal masses such as RCC. On the other hand, RCCs most often occur as a comalignancy. As many as 16–27% of patients with RCC have other synchronous malignancies [2, 5]. In contrast to synchronous cancers, the phenomenon of cancer-to-cancer metastasis is very rare with less than 50 cases described in the literature and most of these metastases were found at autopsy [1]. For successful metastasizing, a cancer requires different characteristics, such as the ability of invasion and dissemination and the appropriate tissue to thrive and grow. It is known that different cancers metastasize to different tissues or organs and this tissue-specific pattern of spread is called tropism [6]. Breast cancer commonly spreads to bone, the lungs, the brain and if abdominal metastasis occur then most often in the liver and/or spleen. Breast cancer metastasizing to the kidneys is almost nonexistent, so the high blood circulation due to the rich vasculature will not be the only decisive factor for metastasis to RCC. Nevertheless, the most frequently described recipient tumor for cancer-to-cancer metastasis is RCC, which is found in up to 65% of the cases with cancer-to-cancer metastasis [3]. The well-preserved stroma, a high content of glycogen and lipid-rich cells and the lower immunological competence of RCC may be more responsible for this predisposition [1, 2, 5]. The metastatic features of RCC and breast cancer, especially of metastasis to the bone, are similar. Therefore, this may be another factor that favors coexistence [7].

A PubMed literature search revealed nine cases of metastasis of a breast cancer into RCC. The six cases reported in which tumors were resected during the lifetime and the present case are presented in detail in Table 1. The present case is one of three reported cases with simultaneous metastasis from breast cancer into RCC. A high breast cancer stage (T2 or more) seems to be responsible for the metastasis. The hormone receptor status was variable among cases. The HER-2 status was negative in the previous series, if specified. In the present case the HER-2 status was positive and both breast cancer tumors had Ki67 levels of over 70%. This case is the only one in a patient with bilateral breast cancer. In contrast to the large breast cancer metastasis described by Begara Morillas et al. [11]and the multiple foci in the other cases, only a small metastasis (0.5 cm) was found in the RCC. Mastectomy was performed in nearly all cases. Information on the outcome of the reported cases was not available in all of the cases but palliative therapy determined the outcome [1, 8–12].

**Table 1:** Survey of case reports on breast cancer metastasis to renal cell carcinoma with diagnosis during life

Author	Interval C–M (years)	Age (years)	Breast cancer							Metastasis sites	Outcome
			Tumor type	TNM	G	ER	PR	HER-2	Ki-67		
Begara Morillas et al. [11]	4	50	No	No	No	No	No	No	No	RCC, bone	Alive 6 months after nephrectomy
Van Wynsberge et al. [10]	6	64	Ductal	T3N1M0	2	−	−	No	No	RCC, lung, liver, and bones	Not specified
Möller et al. [1]	2	62	Ductal	T4N3M0	3	+	+	−	No	RCC, pleura and scalp	Death 10 months after nephrectomy
Huo et al. [12]	4	43	Ductal	T2N0M1	no	+	+	−	20%	RCC, liver, mediastinum	After 3 months disease progression—further treatment was refused
Ulamec et al. [8]	0	60	Ductal^a^	T4N2M1	3	+	+	−	22%	RCC	Without recurrence for 18 months
Perrin et al. [9]	0	49	Ductal	T4N1M1	3	−	−	−	No	RCC, lung and bone	Not specified
Present case	0	79	Ductal bilateral	Right: T2N3M1	3	+	+	+	80%	RCC and bone	Death 3 years after diagnosis by multimorbidity
				Left: T2N0M1	2	+	+	+	70%		

*Interval C–M* interval between breast cancer diagnosis and metastasis to RCC, *G* grading, *ER* estrogen receptor, *PR* progesterone receptor, *HER-2* human epidermal growth factor receptor 2, *Ki 67* proliferation marker, *RCC* clear cell renal cell carcinoma, + positive, − negative, *no* no further information, *TNM* classification of tumor, lymphe nodes, metastasis

^a^With neuroendocrine differentiation

The literature data and this case illustrate that screening for metastases in patients presenting with cancer may lead to the detection of secondary cancers. Suspected secondary lesions should be biopsied to distinguish between metastatic and synchronous cancers. Even if finding a cancer-to-cancer metastasis is a seldom event, it should be considered especially in high stage cancer. In the present patient the finding of breast cancer metastasis in RCC was clinically important to define treatment (curative vs. palliative) goals. Finally, investigation of cancer-to-cancer metastases, even if rare, could provide clues about tumor biology and behavior. Research in this special area could sustain the development of new therapies or detection methods for metastases.
